# The impact of comprehensive medical system reform on rural household economic resilience

**DOI:** 10.3389/fpubh.2026.1928919

**Published:** 2026-10-15

**Authors:** Tianshu Zhang, Jianguo Dong

**Affiliations:** School of Public Administration, Inner Mongolia University of Finance and Economics, Hohhot, China

**Keywords:** comprehensive medical system reform, difference-in-differences, financial vulnerability, medical burden, rural household economic resilience

## Abstract

**Introduction:**

Strengthening the capacity of rural families to withstand health-related economic shocks is critical to consolidating poverty alleviation achievements and preventing large-scale relapse into poverty. However, existing studies pay limited attention to the resilience-shaping effects of the medical security system as a fundamental social policy, especially among rural households.

**Methods:**

Using data from the China Family Panel Studies (CFPS) from 2012 to 2022, this paper employs a single-period difference-in-differences method to empirically examine the impact of comprehensive medical system reform on rural household economic resilience and its underlying mechanisms.

**Results:**

The results indicate that the reform significantly enhances rural household economic resilience, and this finding remains robust across a series of robustness checks. Mechanism analysis suggests that the reform may enhance resilience by reducing medical burden and alleviating financial vulnerability. Heterogeneity analysis further shows that these effects are more pronounced among high-income families and those with higher levels of social trust.

**Discussion:**

This study expands the evaluative horizon of medical security system reforms in terms of economic outcomes and offers empirical evidence to support the deepening of medical reform and the prevention of poverty induced by illness.

## 1 Introduction

Health is a core component of human capital (1, 2) and a fundamental prerequisite for individuals to participate in economic activities and enhance household welfare. The “Healthy China 2030” Planning Outline explicitly calls for prioritizing rural and grassroots areas, advancing the equalization of basic public health services, and elevating the reduction of urban–rural health disparities to a national strategic goal. In 2025, the “Opinions on Further Safeguarding and Improving People’s Livelihood and Addressing the Urgent, Difficult, and Anxious Concerns of the Public” reaffirmed the need to strengthen the risk resilience of low-income populations and urged the expansion and decentralization of high-quality medical and health resources toward a more balanced regional distribution. Nevertheless, rural China has long been confronted with structural challenges, including inadequate medical resource supply and insufficient coverage. When hit by health shocks, rural households often lack effective risk-buffering mechanisms and are vulnerable to falling into the trap of “falling into or returning to poverty due to illness.” In this context, investigating how to bolster the economic capacity of rural families to withstand health-related risks carries both substantial theoretical value and practical significance for solidifying poverty alleviation outcomes and preventing a large-scale resurgence of poverty.

In recent years, the concept of household economic resilience has garnered growing academic interest. It refers to the capacity of households to withstand external shocks that may undermine their well-being and to avoid falling into poverty under uncertain conditions (3). Unlike conventional welfare measures such as income and consumption, economic resilience dynamically captures households’ adjustment and recovery processes before and after shocks, offering a novel lens for evaluating the poverty-reduction effects of policies. Scholars have explored various dimensions of its measurement and determinants. From a digital finance perspective, Yang et al. (4) and Si et al. (5) identified a positive effect of digital financial literacy on rural household economic resilience. Zhang and Jiao, drawing on agricultural insurance literature, confirmed the role of risk transfer instruments in enhancing farmers’ economic resilience (6). Sun et al. further incorporated urban–rural integration development into the analytical framework, revealing how factor mobility and industrial convergence empower rural household resilience (7). While these studies approach the core question of resilience enhancement from diverse angles, existing literature predominantly concentrates on economic or industrial policy impacts, with insufficient attention paid to the resilience-shaping effects of the medical security system as a fundamental social policy.

The comprehensive medical system reform offers a valuable policy setting for examining the influence of social security on household economic resilience. Between 2015 and 2016, the State Council successively launched 11 provincial-level pilot projects for comprehensive medical reform, with the goal of mitigating the enduring challenges of “difficult and expensive medical care” for rural residents through coordinated institutional integration (8). A growing body of literature has evaluated the policy effects of these reforms from multiple angles. Lu et al. found that comprehensive medical reform effectively alleviates household financial vulnerability (9) and facilitates the upgrading of household consumption structures (10). Wang et al. (11) and Wang (12) provided evidence that the reform has eased both access-to-care and cost-related burdens. Similarly, Sun et al. reported that the reform improved residents’ satisfaction with medical services and their perceptions of healthcare quality, while also effectively reducing medical expenses (13).

While the studies above offer multidimensional insights into household economic resilience and comprehensive healthcare reform, thereby establishing a useful foundation for this paper, several gaps remain in the existing literature. First, research on household economic resilience has predominantly centered on economic or industrial policies, with comparatively limited attention given to policy instruments anchored in the social security system. Second, evaluations of comprehensive healthcare reform have largely targeted the general population, overlooking the relatively vulnerable subgroup of rural families. Third, there is a notable scarcity of empirical studies specifically addressing the pilot policies of comprehensive healthcare reform, leaving policy practice insufficiently supported by evidence. To address these gaps, this paper draws on multi-period data from the China Family Panel Studies (CFPS) spanning 2012 to 2022 and employs a single-period difference-in-differences approach to identify the impact and transmission pathways of comprehensive medical system reform on rural household economic resilience. The marginal contributions of this study are threefold. First, it integrates comprehensive healthcare reform and rural household economic resilience into a unified analytical framework, thereby enriching the evaluative dimensions of reform effectiveness. Second, by focusing specifically on rural families, a group characterized by limited medical resources and weak risk-coping capacity, it provides micro-level evidence on the policy effects of the new healthcare reform in rural contexts. Third, it examines two distinct transmission channels through which the reform enhances rural household economic resilience, namely medical burden and financial vulnerability, thereby strengthening the empirical basis for understanding how healthcare security systems affect household welfare.

## 2 Theoretical analysis and research hypotheses

### 2.1 Comprehensive medical system reform and rural household economic resilience

The impact of comprehensive medical system reform on the economic resilience of rural households can be analyzed from two perspectives: health capital theory and poverty trap theory.

On the one hand, health, as a key dimension of human capital, directly affects individuals’ labor supply decisions and production efficiency through its stock level. Health capital theory regards health as a durable capital good with dual attributes of consumption and investment. It not only generates direct utility but also increases future income by extending healthy working time and improving work efficiency (14). However, rural families face a practical challenge in that disease shocks often create a two-way squeeze on their income and expenditure structure. Illness not only reduces labor force participation and income-generating capacity but also generates additional rigid medical expenses. The comprehensive medical system reform has systematically alleviated this predicament from both the supply and demand sides through the coordinated linkage of healthcare services, medical insurance, and pharmaceuticals. On the supply side, the establishment of a hierarchical diagnosis and treatment system and the strengthening of primary healthcare capacity have lowered the barriers for rural residents to access timely and effective services (15). On the demand side, reforms in medical insurance payment methods and the enhancement of benefit levels have directly curbed the growth of total medical expenditures (16). These measures help preserve the health capital stock of rural households, reduce the loss of working time and income caused by health impairment, and strengthen households’ ability to maintain consumption levels and restore economic functioning after risk events, thereby enhancing their overall economic resilience. Moreover, beyond reducing direct medical expenditures, the reform also alleviates labor supply distortions induced by health risks. Wei and Li demonstrate that improved portability of health insurance encourages rural labor migration, suggesting that comprehensive medical reform may further bolster household economic resilience through increased labor market participation and income diversification (17).

On the other hand, the asset-based poverty trap theory suggests that when a household’s asset stock falls below a certain critical threshold, it becomes trapped in a low-level equilibrium and struggles to escape through its own accumulation efforts (18). Disease shocks act as a key mechanism that triggers asset dissipation and perpetuates poverty traps (19). Owing to inadequate risk protection, rural families facing major illnesses are often compelled to sell off assets or take on debt to cover rigid expenses. This process erodes the asset base upon which households rely for long-term development, transforming temporary health shocks into persistent economic hardship. By integrating urban and rural medical insurance schemes and raising the effective reimbursement rate, the comprehensive medical system reform has established a financial risk buffer for rural households. This reduces the likelihood that families will be forced to liquidate assets or interrupt productive investments, thereby providing a solid institutional foundation for preserving and strengthening their economic resilience. Based on the above analysis, this paper proposes hypothesis H1:

*H*1: Comprehensive medical system reform can enhance the economic resilience of rural families

### 2.2 The impact mechanism of comprehensive medical system reform on rural household economic resilience

The unexpected expenditure theory posits that household expenditures can be divided into two categories, namely expected and unexpected expenditures (20). The former are incorporated into routine household budget planning and can be covered by stable income streams. The latter, due to their sudden and unpredictable nature, often exceed households’ current payment capacity, forcing them to cut back on other basic consumption and draw on precautionary savings, thereby severely eroding their economic safety margin. Owing to low income levels and limited liquidity reserves, rural households are particularly vulnerable when facing unexpected expenses such as disease shocks, and their financial buffer space is extremely constrained. The comprehensive medical system reform has raised the effective reimbursement rate for insured rural residents through the coordinated efforts of medical services, insurance, and pharmaceuticals, directly reducing the scale of unexpected expenditures triggered by illness. Meanwhile, the hierarchical diagnosis and treatment system encourages rural residents to seek initial care at primary facilities, thereby avoiding unnecessary cost burdens arising from uninformed choices of higher-level hospitals. These institutional arrangements effectively mitigate the sudden impact of unforeseen medical expenses on household budgets, enabling families to maintain their basic consumption levels when confronted with health risks. This, in turn, supports the stabilization and enhancement of household economic resilience. Based on this, this paper proposes hypothesis H2:

*H*2: Comprehensive medical system reform can enhance the economic resilience of rural households by reducing healthcare burden

The enhancement of household economic resilience through comprehensive medical system reform is not limited to the direct reduction of current medical expenditures; it also exerts a deeper influence by stabilizing households’ future expectations and optimizing their asset allocation structures. Keynes’s precautionary motive in liquidity preference theory illuminates the behavioral rationale behind individuals’ tendency to hold cash in response to uncertain future expenditures (21). Rural households, characterized by substantial income volatility and limited social protection, face dual uncertainties associated with health risks. Even in the absence of actual shocks, they tend to passively hold highly liquid assets as a precaution, which results in a short-term orientation in asset allocation and crowds out resources that could otherwise be directed toward long-term productive investment and human capital accumulation. By integrating urban and rural medical insurance schemes and raising reimbursement rates, the comprehensive medical system reform has conveyed credible risk-sharing signals to rural households, thereby reducing their perceived uncertainty regarding future medical expenses. This, in turn, weakens their precautionary saving motivation and releases locked household funds for more productive uses. In addition, medical insurance lowers the probability that families will resort to high-interest borrowing in response to sudden illness, alleviates the persistent erosion of asset accumulation caused by financial vulnerability, and ultimately strengthens household economic resilience. Based on this, this paper proposes hypothesis H3:

*H*3: Comprehensive medical system reform can enhance the economic resilience of rural households by alleviating their financial vulnerability

## 3 Research design

### 3.1 Data source

The data in this paper is sourced from six tracking periods of the China Family Panel Studiesfrom 2012 to 2022. CFPS is implemented by the China Social Science Survey Center of Peking University, using a stratified multi-stage probability sampling design, covering the vast majority of provinces and regions across the country, and has good national representativeness. Since the research subjects are rural households, this paper processes the data as follows: (1) excluding urban household samples, (2) removing samples with severe missing values and invalid observations. The final sample includes 13,179 annual observations from 6,205 unique rural households. The treatment group contains 1,225 observations, and the number of control groups is 11,954.

### 3.2 Dependent variable

The dependent variable of this paper is household economic resilience. Drawing on Cissé & Barrett’s moment estimation method (22) and referring to the research practices of Li and Lu (23), the economic resilience of households is quantified as the probability of maintaining per capita consumption levels above the poverty line. The specific calculation steps are as follows:

The first step is to estimate the conditional expectation equation for consumption. We employ a Poisson GLM as a quasi-likelihood approach to estimate the conditional mean, which is appropriate because consumption is non-negative and ensures positive predicted means. The model includes consumption lag terms and household characteristic variables. Considering the S-shaped dynamic characteristics revealed by the multiple equilibrium poverty trap theory, this paper sets the high-order center distance order k of the consumption lag term to 3 (24). The specific model setting is:

$$W_{\mathrm{it}}=\sum_{q=1}^{3}k_{\mathrm{Mq}}W_{\mathrm{it}-1}^{q}+\delta_{M}X_{\mathrm{it}}+\eta_{\mathrm{Mit}}$$ (1)

Among them, W_it_ is the per capita consumption level of household i in year t (taking its logarithm), W_it-1_ is the per capita consumption level lagged by one period, X_it_ is the control variable, η_Mit_ is the random disturbance term, and the subscript M represents the expectation equation. Using the estimation result of Equation 1 and the assumption of zero mean random error term E (η _Mit_)=0, the conditional expectation prediction value of household i in year t is obtained as Equation 2:

$${\hat{\mu }}_{\mathrm{it}}=E[W_{\mathrm{it}}|W_{\mathrm{it}-1},X_{\mathrm{it}}]=\sum_{q=1}^{3}{\hat{k}}_{\mathrm{Mq}}W_{\mathrm{it}-1}^{q}+{\hat{\delta }}_{M}X_{\mathrm{it}}$$ (2)

The second step is to estimate the conditional variance equation of consumption. Following the approach of Just & Pope (25) and Antle (26), using the square term of the residuals obtained in the first step as a proxy variable for the second-order center distance, regression is performed on the consumption lag term and feature variables, as shown in Equation 3:

$${\hat{\eta }}_{\mathrm{Mit}}^{2}\begin{array}{c} =\sum_{q=1}^{3}k_{\mathrm{Vq}}W_{\mathrm{it}-1}^{q}+\delta_{V}X_{\mathrm{it}}+\eta_{\mathrm{Vit}} \end{array}$$ (3)

Following the zero mean assumption E (η Vit) = 0, the conditional variance prediction value of household i in year t is given by Equation 4:

$$\begin{array}{c} {\hat{\mu }}_{2\mathrm{it}}=\sum_{q=1}^{3}{\hat{k}}_{\mathrm{Vq}}W_{\mathrm{it}-1}^{q}+{\hat{\delta }}_{V}X_{\mathrm{it}} \end{array}$$ (4)

The third step is to calculate the economic resilience of the household. Following the analytical framework of Barrett & Constas (27), household economic resilience ρ_it_ is defined as the probability that the per capita consumption level of a household exceeds a specific poverty line threshold $\bar{W}$ :

$$\begin{array}{c} {\hat{\rho }}_{\mathrm{it}}=P(W_{\mathrm{it}}\geq \bar{W})=1-\Phi (\frac{\bar{W}-{\hat{\mu }}_{\mathrm{it}}}{{\hat{\sigma }}_{\mathrm{it}}}) \end{array}$$ (5)

where ${\hat{\mu }}_{\mathrm{it}}$ is the estimated conditional mean, ${\hat{\sigma }}_{\mathrm{it}}=\sqrt{{\hat{\mu }}_{2\mathrm{it}}}$ is the estimated conditional standard deviation, $\bar{W}$ is the log-transformed poverty threshold, and Φ(·) is the cumulative distribution function of the standard normal distribution. To account for price changes between 2012 and 2022, this paper uses the 2010 constant price of 2,300 yuan as the base poverty line and adjusts the threshold for each year using the rural consumer price index (CPI), thereby ensuring cross-period comparability. The resulting poverty line standards for each survey year are 2,625 yuan in 2012, 2,800 yuan in 2014, 2,952 yuan in 2016, 2,995 yuan in 2018, 3,442 yuan in 2020 (28), and approximately 3,535 yuan in 2022 (calculated based on the rural CPI).

### 3.3 Core independent variable

The core explanatory variable of this paper is the comprehensive medical system reform. The pilot reform of China’s comprehensive medical system has been rolled out in multiple batches. In 2015, Jiangsu, Anhui, Fujian, and Qinghai were designated as the first batch of pilot provinces. In 2016, seven additional provinces (regions, municipalities) including Shanghai, Zhejiang, Hunan, Chongqing, Sichuan, Shaanxi, and Ningxia Hui Autonomous Region were added as the second batch, bringing the total number of pilot provinces to 11. Since CFPS data is usually released in even numbered years, and considering the coordinated changes in medical services, insurance, and drug pricing involved in comprehensive healthcare reform, each province usually needs extra time to develop implementation plans, establish cross departmental coordination mechanisms, and introduce specific policies, which have a certain lag. Therefore, following existing research (29), merges these two batches of pilot data, and uniformly considers 2016 as the year of policy implementation.

The specific operation is as follows: first, set the treat variable and mark the pilot province as 1, otherwise it will be 0. Next, construct a post variable and mark regions where the sample survey year is greater than the pilot year as 1, and vice versa as 0. Finally, the interaction term combination (treat x post) is used to obtain the DID variable of this paper, which is the comprehensive medical system reform. The pilot provinces for comprehensive medical system reform are shown in Table 1.

**Table 1:** Pilot province for comprehensive medical system reform.

Category	Pilot period	Province	N
Treatment group	2015	Jiangsu, Anhui, Fujian, Qinghai	1,225
	2016	Shanghai, Zhejiang, Hunan, Chongqing, Sichuan, Shaanxi, Ningxia	
Control group		Other provinces	11,954

### 3.4 Control variables

Referring to the research of Tian et al. (30),the control variables in this paper mainly include mainly include gender, age, marital status, education level and family size.

### 3.5 Mediating variable

The mechanism variables in this paper include medical burden and financial vulnerability. Among them, the medical burden is measured by the proportion of self-funded medical expenses, specifically measured by the proportion of household medical expenses to household nonfood consumption expenses (31). The financial vulnerability is constructed using the financial margin method by referring to Li Bo and Zhu Taihui (32), and its formula is:

$$\begin{array}{c} {\mathrm{FM}}_{\mathrm{it}}={\text{Income}}_{\mathrm{it}}+{\mathrm{LA}}_{\mathrm{it}}-{\mathrm{Exp}}_{\mathrm{it}}-{\text{Unexp}}_{\mathrm{it}} \end{array}$$ (6)

$$\begin{array}{c} {\text{Financial fragility}}_{\mathrm{it}}=1({\mathrm{FM}}_{\mathrm{it}}<0) \end{array}$$ (7)

In Equation 6, FM_it_ represents financial margin. Income refers to the total household income. LA_it_ is a liquid asset. Exp_it_ is the expected expenditure, including daily total household expenses and mortgage payments. Unexp_it_ refers to unexpected expenses and represents household healthcare expenditures. In Equation 7, financial fragility refers to the financial fragility of households. Table 2 presents descriptive statistics of variables.

**Table 2:** Descriptive statistics of variables.

Variable type	Variable	Definition and assignment	Mean	Std. dev.	Min	Max
Explained variable	household economic resilience	Calculated according to Equation 5	0.200	0.016	0.115	0.246
Core explanatory variables	Comprehensive medical system reform	yes = 1; no = 0	0.034	0.181	0.000	1.000
Control variable	Gender	male = 1; female = 0	0.659	0.474	0.000	1.000
	Age	30 years old and above	54.112	11.305	31.000	93.000
	Marriage	yes = 1; no = 0	0.874	0.332	0.000	1.000
	Edu	Primary school and below = 0. Junior high school = 1. High school = 2. College degree or above = 3	0.674	0.842	0.000	3.000
	Familysize	1 person or more	3.903	1.903	1.000	15.000
Mechanism variable	Medical burden	The proportion of household medical expenses to household nonfood consumption income	0.126	0.191	0	1
	Financial fragility	According to Equation 7, it is obtained	0.376	0.484	0	1

### 3.6 Model specification

This paper uses a difference-in-differences to evaluate the impact of comprehensive healthcare reform pilot on household economic resilience. The model is set as follows:

$$\begin{array}{c} Y_{\mathrm{it}}=\alpha +\beta \cdot {\mathrm{DID}}_{\mathrm{it}}+\gamma X_{\mathrm{it}}+\mu_{i}+\lambda_{t}+\in_{\mathrm{it}} \end{array}$$ (8)

In Equation 8, Y_it_ is the dependent variable. DID_it_ is the core explanatory variable. X_it_ is the set of control variables. μ_i_ is the household fixed effect. λ_t_ is the fixed effect of the year. ϵ_i_ is the random error term. The coefficient β is the policy treatment effect that this paper focuses on.

## 4 Empirical analysis

### 4.1 Analysis of regression results

All regressions in this paper control for household and year fixed effects, with standard errors clustered at the provincial level. Given the relatively small number of provincial clusters, we employ wild bootstrap standard errors with 999 replications and Rademacher weights, implemented using the boottest command in Stata, to improve inference reliability. Table 3 reports the benchmark regression results of the comprehensive medical system reform on rural household economic resilience. Column (1) includes only the core explanatory variable, and the coefficient of the comprehensive medical system reform is 0.0054, which is statistically significant at the 1% level. Column (2) further incorporates household-level control variables, and the coefficient is 0.0048, remaining significant at the 1% level. These results indicate that the comprehensive medical system reform significantly enhances the economic resilience of rural households, thereby validating research hypothesis H1.

**Table 3:** Benchmark regression results.

Variable	Household economic resilience	
	(1)	(2)
Comprehensive medical system reform	0.0054*** (0.0014)	0.0048*** (0.0011)
Control variables	N	Y
Household fixed effects	Y	Y
Year fixed effects	Y	Y
R-squared	0.106	0.339
N	13,179	13,179

* * *,* *and *, respectively, indicate *p* < 0.01, *p* < 0.05 and *p* < 0.1. Standard errors are clustered at the provincial level Significance levels are based on wild bootstrap with 999 replications.

### 4.2 Robustness tests

#### 4.2.1 Parallel trend test

The parallel trend assumption is a fundamental prerequisite for identifying causal effects in difference-in-differences models, requiring that the treatment and control groups exhibit similar trends prior to policy implementation. This paper employs an event study approach to test this assumption, using the period before policy implementation as the baseline and generating dummy variables for each period before and after the reform, which are then incorporated into the regression model. Figure 1 presents the results of the parallel trend test. The estimated coefficients for all pre-policy periods are not statistically significant, and their confidence intervals consistently contain zero, indicating that there was no systematic difference in rural household economic resilience between the treatment and control groups before the launch of the comprehensive medical reform pilot. Thus, the analysis does not detect evidence against the parallel trend assumption. The policy effect becomes significantly positive from the current period onward, and the overall effect exhibits an upward trajectory after implementation, suggesting that the economic resilience of rural households in the treatment group improved markedly following the comprehensive medical system reform.

**Figure 1:**
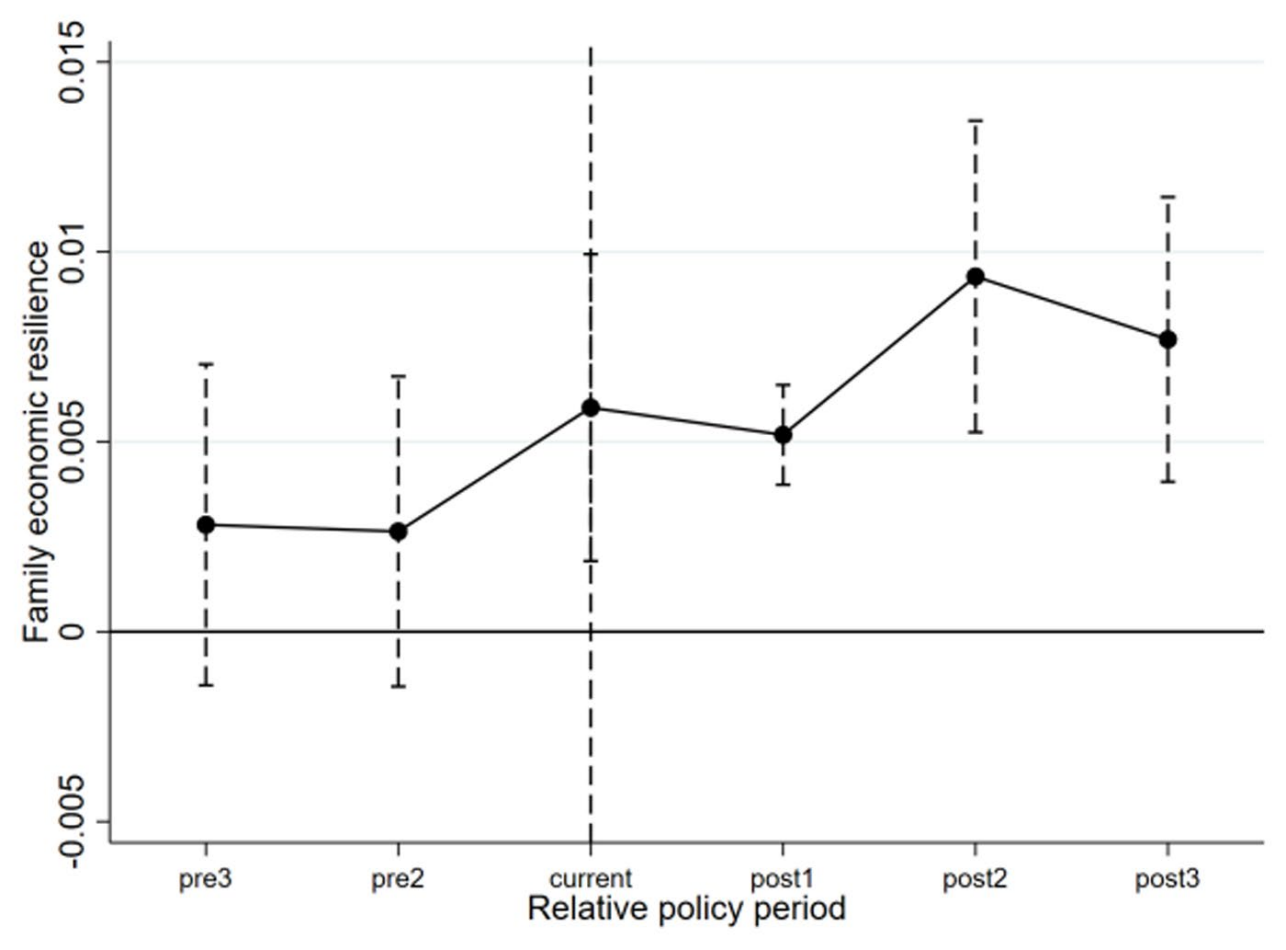
Parallel trend test.

#### 4.2.2 Placebo test

To further rule out the possibility that the significant differences between the treatment and control groups are driven by other unobservable random factors, this study conducts a placebo test. Specifically, we randomly assign provinces as the pseudo-treatment group in each replication, generate a fictitious policy shock variable, and re-estimate the DID coefficient based on this province-level grouping. This procedure is repeated 500 times, and the kernel density distribution of the estimated coefficients is plotted. Figure 2 displays the distribution of the 500 simulated DID coefficients. The results show that the placebo estimates are concentrated around zero and approximately follow a normal distribution, whereas the true policy effect estimated in the benchmark regression lies well outside the central region of this distribution. This confirms that the impact of comprehensive medical system reform on rural household economic resilience is not driven by random factors, and the benchmark regression results are robust.

**Figure 2:**
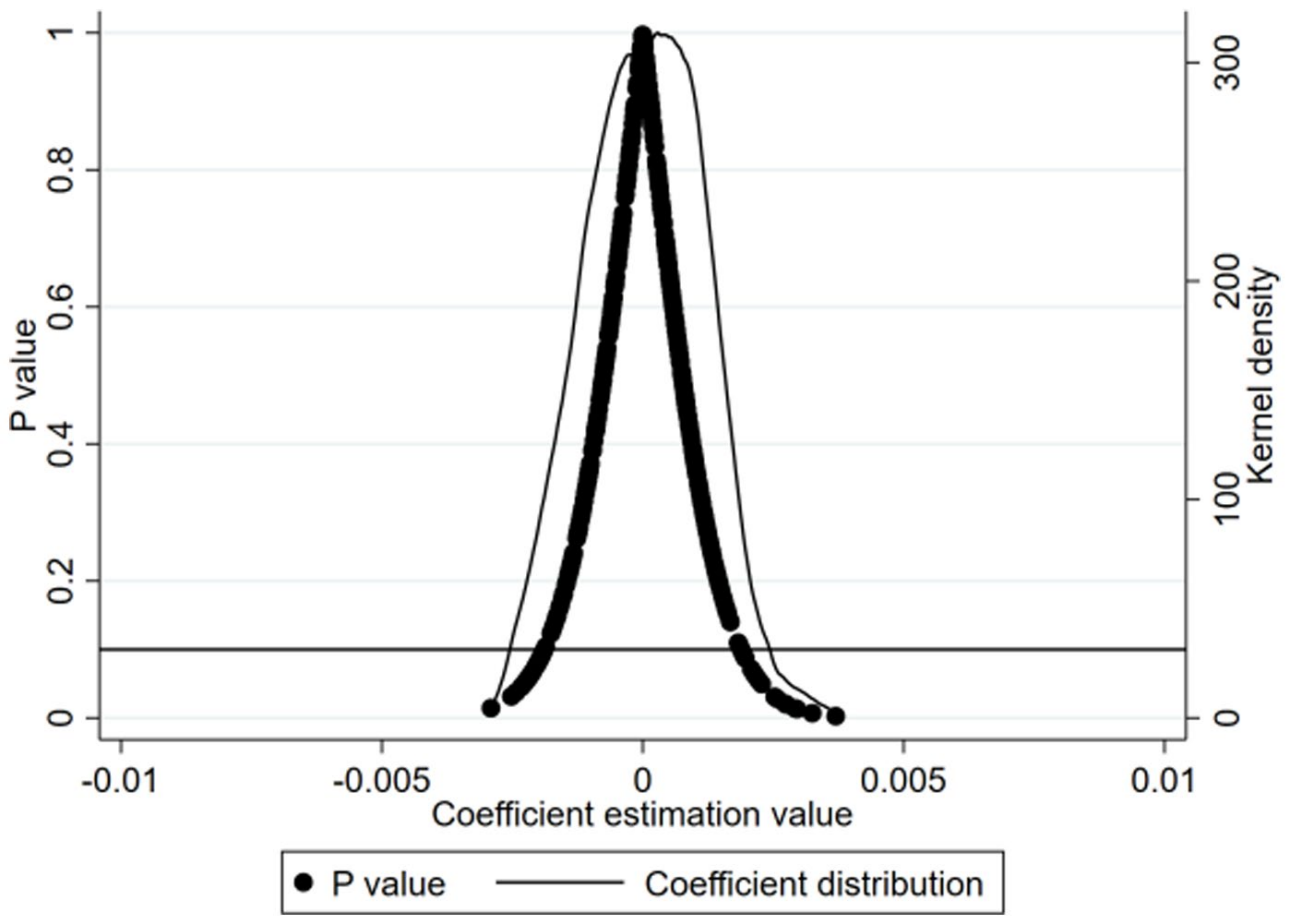
Placebo test.

#### 4.2.3 Replace the dependent variable

To assess whether our findings are sensitive to the choice of poverty threshold, we re-measure household economic resilience using an alternative poverty line set at 40% of the sample median per capita consumption (33) and re-estimate the DID model. As shown in column (1) of Table 4, the coefficient for comprehensive medical system reform remains positive and statistically significant at the 1% level, confirming that our baseline results are not driven by the specific poverty line chosen.

**Table 4:** Replace the dependent variable and single batch pilot inspection.

Variable	Household economic resilience	
	(1)	(2)
Comprehensive medical system reform	0.0048*** (0.0008)	0.0044*** (0.0009)
Control variables	Y	Y
Household fixed effects	Y	Y
Year fixed effects	Y	Y
R-squared	0.339	0.203
N	13,179	13,179

#### 4.2.4 Single batch pilot inspection

Since the comprehensive medical system reform was launched in two batches in 2015 and 2016, while the CFPS survey years are all even-numbered, and given the typical time lag in policy effects, this paper defines both pilot batches as entering the treatment period from 2016 onward in the benchmark regression. To examine whether this treatment approach affects our conclusions, we exclude the first-batch pilot provinces (2015) and re-estimate the model using only the second-batch pilot provinces (2016) as the treatment group. The results are presented in column (2) of Table 4. After excluding the 2015 pilot samples, the coefficient of comprehensive medical system reform is 0.0044, remaining significant at the 1% level. This indicates that merging the two policy pilot batches in the baseline specification does not materially affect the regression results, confirming the robustness of our findings.

#### 4.2.5 Alternative lag structure

To examine whether the construction of the resilience measure is sensitive to the choice of the polynomial order (k) for the lagged consumption terms, we re-estimate the baseline model using alternative specifications with k = 1 (column 1) and k = 2 (column 2). The results are reported in Table 5. The DID coefficients remain significantly positive at the 1% level under both alternative specifications (0.0048 for k = 1 and 0.0053 for k = 2), confirming that our main findings are not driven by the specific choice of k = 3.

**Table 5:** Alternative lag structure.

Variable	Household economic resilience	
	(1)	(2)
Comprehensive medical system reform	0.0048*** (0.0008)	0.0053*** (0.0009)
Control variables	Y	Y
Household fixed effects	Y	Y
Year fixed effects	Y	Y
R-squared	0.192	0.197
N	13,179	13,179

#### 4.2.6 Exclude interference from other policies

During the observation window of this study, the government simultaneously implemented pilot programs for long-term care insurance and public hospital reform, both of which may have influenced the economic resilience of rural households. Although long-term care insurance primarily targets disability care, it can alleviate the medical burden on rural families by reducing home care expenses, thereby potentially enhancing their economic resilience. Likewise, the public hospital reform pilot, by abolishing drug markups and adjusting service prices, directly affects the supply side and price level of medical services, and may also alter households’ actual medical expenditures. Accordingly, to eliminate potential interference from these concurrent policies, we construct policy dummy variables based on the launch dates of long-term care insurance pilots in each city and the announcement timing of public hospital reform pilot lists, and include them in the benchmark regression.

The regression results after controlling for other policy interferences are reported in Table 6. Column (1) adds only the long-term care insurance pilot variable to the benchmark model, and the estimated coefficient for comprehensive medical reform is 0.0051, significant at the 1% level. Column (2) adds only the public hospital reform pilot variable, and the coefficient for comprehensive medical reform is 0.0047, also significant at the 1% level. Column (3) includes both policy variables simultaneously, and the estimated coefficient for comprehensive healthcare reform is 0.0050, remaining significant at the 1% level. These results indicate that, whether controlling for the potential impact of long-term care insurance and public hospital reform pilots separately or jointly, the positive effect of comprehensive medical system reform on rural household economic resilience remains robust, further reinforcing the reliability of our benchmark conclusions.

**Table 6:** Exclusion of other policy interference.

Variable	Household economic resilience		
	(1)	(2)	(3)
Comprehensive medical system reform	0.0051*** (0.0009)	0.0047*** (0.0009)	0.0050*** (0.0008)
Long term care insurance	Y	N	Y
Reform of public hospitals	N	Y	Y
Control variables	Y	Y	Y
Household fixed effects	Y	Y	Y
Year fixed effects	Y	Y	Y
R-squared	0.205	0.206	0.206
N	13,179	13,179	13,179

#### 4.2.7 Shorten the time window

Given that the COVID-19 outbreak in 2020, as a sudden public health crisis, exerted a systematic exogenous shock on rural households’ employment and income generation, which may interfere with the accurate identification of the comprehensive medical reform policy effects, we exclude data from 2020 and 2022 and shorten the sample window to 2012–2018, then re-estimate the DID model. The results in column (1) of Table 7 show that the coefficient of the comprehensive healthcare reform policy is 0.0044, remaining significantly positive at the 1% level. This indicates that after excluding the special impact of the pandemic, the positive effect of the comprehensive medical system reform on rural household economic resilience remains robust.

**Table 7:** Shortening policy windows and deleting special samples.

Variable	Household economic resilience	
	(1)	(2)
Comprehensive medical system reform	0.0044*** (0.0008)	0.0053*** (0.0009)
Control variables	Y	Y
Household fixed effects	Y	Y
Year fixed effects	Y	Y
R-squared	0.221	0.205
N	9,796	12,891

#### 4.2.8 Delete special samples

Given the substantial differences in medical resource density and economic development levels between China’s four municipalities directly under the central government and ordinary provinces, the development environment and public service capacity in rural areas of these municipalities may differ considerably from those in other provinces. To avoid potential interference from these special factors, this paper follows existing research (34) and re-estimates the DID model after excluding samples from the four municipalities. The results in column (2) of Table 7 show that the DID coefficient is 0.0053, remaining significantly positive at the 1% level, which further strengthens the robustness of the benchmark conclusions.

## 5 Heterogeneity analysis

### 5.1 Income level

Income level is a core factor influencing a family’s ability to cope with risks. Rural households with different income levels differ considerably in their risk reserves and strategies for responding to shocks. As a result, the effect of comprehensive medical system reform on enhancing household economic resilience may be heterogeneous. To test this conjecture, we divide the full sample into low-income and high-income groups based on the median of total household income and conduct separate DID estimations for each group.

Table 8 reports the regression results grouped by income level. Column (1) presents the findings for low-income families, where the DID coefficient is 0.0100 and statistically significant at the 1% level. Column (2) shows the results for high-income families, with a DID coefficient of 0.0082, also significant at the 1% level. At the same time, this paper further conducts a between-group difference test by constructing an interaction term. As shown in column (3) of Table 8, the coefficient of the interaction term between DID and the high-income group is 0.0056, which is statistically significant at the 1% level. This indicates that the policy effect for high-income households is significantly greater than that for low-income households, suggesting that comprehensive medical reform has a more pronounced effect on enhancing the economic resilience of higher-income families.

**Table 8:** Analysis results of income level heterogeneity.

Variable	Household economic resilience		
	(1)	(2)	(3)
Comprehensive medical system reform	0.0100*** (0.0013)	0.0082*** (0.0008)	0.0059*** (0.0010)
DID*high income			0.0056*** (0.0006)
	
Control variable	Y	Y	Y
Household fixed effects	Y	Y	Y
Year fixed effects	Y	Y	Y
N	6,595	6,584	13,179
R-squared	0.173	0.297	0.199

### 5.2 Social trust level

Social trust, as a core dimension of social capital, reflects the degree to which residents trust social institutions and interpersonal relationships. In rural areas, the level of social trust directly affects a family’s access to information and its willingness to cooperate with public policies. In the context of medical services, families with higher levels of social trust are more likely to have confidence in medical institutions and health insurance policies, to actively utilize healthcare resources, and thereby to fully benefit from policy dividends. Consequently, the effect of comprehensive healthcare reform on enhancing the economic resilience of rural households may vary with the level of social trust. This paper uses the “level of trust in strangers” from the CFPS questionnaire as a proxy variable for social trust. The sample is divided into low-trust and high-trust groups based on the median value, and DID estimation is performed separately for each group. Between-group differences are tested by constructing interaction terms. The results are presented in Table 9.

**Table 9:** Heterogeneity analysis results of social trust level.

Variable	Household economic resilience		
	(1)	(2)	(3)
Comprehensive medical system reform	0.0027*** (0.0008)	0.0058*** (0.0016)	0.0031*** (0.0008)
DID*high trust			0.0030*** (0.0007)
Control variable	Y	Y	Y
Household fixed effects	Y	Y	Y
Year fixed effects	Y	Y	Y
N	6,950	6,229	13,179
R-squared	0.174	0.230	0.206

The results indicate that the comprehensive healthcare reform exerts a significant positive effect on the economic resilience of rural households in both the low-trust group (column 1) and the high-trust group (column 2), with DID coefficients of 0.0027 and 0.0058, respectively, both significant at the 1% level. The interaction term test further shows that the coefficient for the interaction between DID and the high-trust group is 0.0030, significant at the 1% level, indicating that the policy effect for high-trust families is significantly larger than that for their low-trust counterparts.

## 6 Mechanism analysis

### 6.1 Medical burden

Theoretical analysis suggests that the comprehensive medical system reform has directly reduced the scale of unexpected medical expenses caused by illness by raising the effective reimbursement rate and guiding patients toward primary care, thereby alleviating the medical burden on rural families. To verify this potential path, this paper measures medical burden as the proportion of out-of-pocket medical expenses, calculated as the ratio of household medical expenditures to household non-food consumption income. A higher value indicates a heavier medical burden. Column (1) of Table 10 reports the test results for the medical burden. The coefficient of the comprehensive medical system reform is −0.0739, significant at the 1% level. In addition, we further examine the impact of medical burden on household economic resilience, as shown in column (3) of Table 10. The coefficient of medical burden is −0.0063, also significant at the 1% level. These results indicate that the comprehensive medical system reform can reduce the proportion of out of pocket medical expenses for rural households, alleviate the direct pressure of disease shocks on household budgets, and help improve household economic resilience to a certain extent. This is consistent with the potential path proposed in the theoretical analysis section, therefore the hypothesis H2 in this study is validated.

**Table 10:** Mechanism analysis results.

Variable	Household economic resilience			
	(1)	(2)	(3)	(4)
Comprehensive medical system reform	−0.0739*** (0.0197)	−0.1367*** (0.0322)	0.0042*** (0.0011)	0.0041*** (0.0008)
Medical burden			−0.0063*** (0.0007)	
Financial Vulnerability				−0.0051*** (0.0003)
Control variables	Y	Y	Y	Y
Household fixed effects	Y	Y	Y	Y
Year fixed effects	Y	Y	Y	Y
R-squared	0.009	0.017	0.214	0.253
N	11,291	13,038	11,291	13,038

### 6.2 Financial vulnerability

Theoretical analysis suggests that comprehensive medical system reform can enhance household economic resilience by stabilizing expectations regarding healthcare security, reducing future uncertainty, and alleviating financial vulnerability. To verify this potential path, this paper employs the financial margin approach to measure household financial vulnerability. This indicator identifies whether a household’s financial buffer, defined as total household income plus liquid assets minus expected and unexpected expenditures, is negative. If the financial margin falls below zero, the household is considered financially vulnerable and assigned a value of 1; otherwise, it is 0. Column (2) of Table 10 reports the test results for the financial vulnerability. The coefficient of the comprehensive medical system reform is −0.1367, significant at the 1% level. Furthermore, we examine the impact of financial vulnerability on household economic resilience, as shown in column (4) of Table 10. The coefficient of financial vulnerability is −0.0051, also significant at the 1% level. These findings suggest that comprehensive medical system reform can reduce the likelihood of economic vulnerability in rural households, and helping to improve their economic resilience to some extent. This is consistent with the potential path proposed in the theoretical analysis section, therefore the hypothesis H3 in this study is validated.

## 7 Conclusion and recommendations

Based on six waves of data from the China Family Panel Studies (CFPS) spanning 2012 to 2022, this paper employs a single-period difference-in-differences method to empirically examine the impact and mechanisms of comprehensive medical system reform on rural household economic resilience. The main conclusions are threefold. First, the comprehensive medical system reform significantly improves the economic resilience of rural households, and this result remains robust across a series of robustness checks. Second, mechanism analysis suggests that the reform may enhance resilience by reducing medical burden and alleviating financial vulnerability, which is consistent with the potential pathways indicated by the theoretical analysis. Third, heterogeneity analysis indicates that the positive effect of the reform on rural household economic resilience is more pronounced among high-income families and those with higher levels of social trust. Based on these findings, this paper proposes the following policy recommendations:

First, we should continue to deepen the comprehensive medical system reform by further expanding policy coverage and enhancing benefit generosity. It is essential to systematically synthesize the reform experiences of the first and second batches of pilot provinces, with particular focus on extracting effective practices in medical insurance payment reform, the development of hierarchical diagnosis and treatment systems, and the strengthening of primary healthcare service capacity. These experiences should be distilled into replicable and scalable institutional models for orderly extension to non-pilot provinces. In this process, special attention should be paid to the specific characteristics of rural areas, steadily increasing the effective reimbursement rate for rural residents’ medical expenses, narrowing the urban–rural gap in medical insurance benefits, and effectively reducing the medical burden on rural families.

Second, we should improve targeted support measures to ensure that all families can effectively benefit from the reform. The heterogeneity analysis in this paper indicates that the marginal improvement effect of comprehensive healthcare reform on economic resilience is more pronounced among higher-income families, suggesting that low-income households may still face barriers in fully accessing and utilizing the policy benefits. Therefore, the institutional framework should be further refined to enable vulnerable groups to share in the gains of the reform. Specifically, we should optimize the coordination among basic medical insurance, critical illness insurance, and medical assistance, lower the deductible thresholds for medical assistance targeted at low-income rural families, and explore differentiated cost-reduction arrangements based on their income levels. In addition, policy outreach and service guidance should be strengthened for low-income and low-trust families to enhance their awareness of and ability to utilize available benefits, thereby weaving a robust safety net that prevents poverty caused by illness and protects against relapse into poverty.

Third, we should strengthen policy outreach and service guidance for households. The heterogeneity analysis reveals that families with lower levels of social trust tend to utilize policy benefits less effectively than their higher-trust counterparts. To address this, it is advisable to leverage grassroots medical service networks, such as village clinics and township health centers, to integrate medical insurance policy advocacy into their routine functions. Using concise and accessible promotional materials and straightforward communication methods, door-to-door outreach should be conducted. Particular emphasis should be placed on building trust in the medical insurance system through transparent information disclosure and direct interpersonal engagement. At the same time, the medical insurance reimbursement process should be streamlined by implementing one-stop settlement services, thereby reducing administrative burdens and information barriers that disproportionately affect low-trust families. These efforts will help ensure that policy resources reach all households effectively, regardless of their varying levels of social trust.

Fourth, we should improve the monitoring and early warning system for financial risks facing rural households. The mechanism analysis in this paper shows that comprehensive healthcare reform enhances household economic resilience partly by alleviating financial vulnerability, suggesting that financial vulnerability can serve as a forward-looking indicator for identifying the risk of falling back into poverty. It is therefore recommended to incorporate a household financial health assessment module centered on financial margins into the existing dynamic monitoring system for low-income populations. For rural households identified as financially vulnerable, a graded early warning mechanism should be implemented, and policy instruments such as medical assistance and temporary subsistence subsidies should be deployed comprehensively for early intervention. Such measures would help families build necessary financial buffers before experiencing health shocks, thereby strengthening their resilience to uncertain disturbances.

## 8 Limitations and future research

Although this study employs a range of rigorous methods for analysis and robustness checks, several limitations remain. First, the biennial frequency of CFPS data and the inherent lag of policy effects require merging the two pilot batches and treating 2016 as the common policy year. While robustness checks support the reasonableness of this specification, it may not fully capture potential cohort-specific treatment effects. Second, the income-based heterogeneity analysis relies on household income, which may itself be affected by the reform, and this analysis should therefore be regarded as exploratory. Third, the mechanism analysis may not fully establish causal mediation, and the results are accordingly presented as evidence consistent with the potential pathways rather than definitive proof of causal mediation. Future research could further explore the long-term dynamic effects of the reform and incorporate more granular data to identify cohort-specific treatment effects.

## Data Availability

Publicly available datasets were analyzed in this study. This data can be found at: https://cfpsdata.pku.edu.cn/.
